# Managing bioanalytical studies during the COVID-19 pandemic: an overview

**DOI:** 10.4155/bio-2020-0082

**Published:** 2020-04-14

**Authors:** Peter Pruim, Naomi Teekamp

**Affiliations:** ^1^PRA Health Sciences, Early Development Services, Bioanalytical Laboratory, Assen, The Netherlands

**Keywords:** bioanalysis, COVID-19, GLP, study management

In this time of crisis due to the global COVID-19 pandemic, most people are staying at home to slow down the spread of the virus and the resulting pressure on their healthcare systems. This is either because of a government-ordered lockdown or self-imposed quarantine. The extent of the measures differ country-to-country and state-to-state. Where companies are allowed to stay operational or where bioanalytical laboratories are regarded part of the essential healthcare infrastructure, bioanalytical studies can still continue. Most laboratories have reduced the presence of office staff in their facilities to the absolute minimum. This includes management, project managers, study directors and supporting staff. In order to ensure continuation of the bioanalytical work, especially those which directly impact the safety of volunteers and patients in clinical studies and those which are done as part of the fight against the COVID-19 pandemic, the laboratory analysts and essential facility staff are allowed to come into the laboratory.

In this editorial, we would like to give some attention to the measures taken to minimize the chance to spread the COVID-19 virus and the impact this has on the analysts working in our laboratory and the study managers. The following roles of the study manager apply: study director (SD) or principal investigator (PI) in studies with a GLP claim or the project manager (PM) in a study where samples from a clinical trial are analyzed good clinical laboratory practice (GCLP) as single point of control.

## General measures

Next to general preventive measures such as disinfection of the hands upon arrival in the building, we have done everything possible to ensure the recommended social distancing of at least 1.5 m in our laboratory in Assen, The Netherlands. As samples are always considered potentially hazardous and are handled as such, no additional preventive measures have been implemented for handling samples from volunteers that are not suspected to be infected with COVID-19. However, samples from volunteers suspected to be infected with COVID-19 are analyzed in our BSL-2 biohazard laboratory.

## Impact on the laboratory analysts

During the critical steps of the sample preparation process at the bench in a laboratory, it can become crowded at times, so work shifts have been initiated by the analysts to decrease the number of colleagues working at the bench at the same time. The number of work places at each bench has been limited and plexiglass plates are placed between the work spaces for protection. Contact between the departments is minimized by the use of separate entrances and staircases.

Now that most office staff are working from home, the analysts are spread out over the building. This includes not just the normal administration rooms of the analysts, but also in meeting rooms and offices that are currently not in use, to perform data processing. This allows us to keep a safe distance. Awkward situations may arise in the corridors, which are 1.5 m wide. Colleagues stand on the look out until the corridor is empty so that they can walk to the laboratory or mass spectrometry instruments without running into another colleague.

In a normal situation, a lot of time is spent together behind a computer evaluating results. As more distance is needed now, online screen sharing or opening the same file is the most used alternative. Of course, where it can be scheduled, data processing or review activities are also performed from home. Even though it can be inconvenient at times, the extra space has some additional benefits: the undisturbed working allows more focused activity. Although the work environment and the interaction within the project team has changed, the quality and batch acceptance rate are at least as good as before the lockdown started.

## Impact on the study managers

When one considers working from home, the question arises if the role of single point of control can be fulfilled remotely, without compromising on quality. This is a topical subject, as the study managers currently working from home, forced by the circumstances, are exploring the borders of remotely managing bioanalytical studies. Communication with the sponsors and clinical sites can be done from home as efficiently as from the laboratory. The most critical and the biggest change in the working situation is in the contact with the project team in the laboratory. With a laptop, internet and network access, the home office is easily set up. Add a phone or a headset and the difference with the normal office is just its location. Using all communication available – phone, video- and tele-conferencing, e-mail and instant messaging – not only the usual business with external contacts can continue, also the daily contact with colleagues is easily established. Although it takes some getting used to spending several more hours on the phone than usual, and occasionally a crash course in the use of Information and Communication Technology (ICT) tools is needed, within a couple of days we realized that a surprising number of things that normally require some kind of written approval can also be documented digitally. Some of us even wondered why we have not used all these digital solutions before. For example, for items of the laboratory process that are not yet in our electronic laboratory notebook, the evaluations of data may not be done in the paper laboratory notebook, but through electronically signed notes-to-file. Scanned copies of relevant records are attached to the notes-to-file for evidence that all results are taken into account. With this approach, documentation can still be done in a timely manner and the quality of the study is assured.

From the above it seems that the single point of control is achievable remotely. However, in our opinion, a one size fits all approach from home would not work due to the variety of studies ongoing in the laboratory, for example, different degrees of regulation may be applicable. How would that affect working remotely? According to the Organisation for Economic Co-operation and Development (OECD) GLP principles [1], the study director has the overall responsibility for the conduct of the study and for its final report at the test facility or the principal investigator at the test site. The phrase “responsibility for the overall conduct of the study and for its final report” may be interpreted in a broader sense for those studies where the Study Director may be geographically remote from parts of the actual experimental work.

For GCLP studies [2,2] the need for a project manager at the laboratory is not described in detail. Although regulators do not specifically require an on-site single point of control, it is for the majority of the studies most practical to have one. For those studies a balance between taking the responsibility over the study and being a responsible citizen and staying at home needs to be established.

We therefore decided to make a distinction between studies with a GLP claim and bioequivalence studies and all other studies. For GLP and bioequivalence studies, we decided that the SD, PI or PM should be considered essential staff for the study. The SD, PI or PM should therefore come into the laboratory regularly to evaluate data, discuss progress, deviations and investigations in a timely manner and on-site if needed. The SD, PI or PM will leave the laboratory directly after that. For GCLP (other than bioequivalence studies) the physical presence of the PM is not needed so very often and most evaluations and discussions can be done in a timely manner remotely through the digital means available. For these studies, we decided that the PM can come in to the laboratory once a week for progress discussions with the project teams, if this is required, in addition to the remote discussions. This distinction gives the PM more time to work uninterrupted at home, allowing for more time for the review of reports and documentation.

## Concluding remarks

In our experience, this approach works satisfactorily and allows us to continue the development of medication while keeping a responsible distance from each other. Needless to say, a bit more flexibility than usual is needed from all involved as many colleagues need to divide their time between their work and their personal commitments such as taking care of their children and home-teaching activities. The situation in our laboratory is probably not very different to many other bioanalytical laboratories across the world at this point in time. This again shows the commitment of bioanalytical scientists toward the improvement of patient lives.

This pandemic will end and before we go back to our usual lives and jobs, let us learn from our current experiences in how we can use the available technology to work from home, efficiently and without compromising on quality. The flexibility that all of us are displaying now may help us focus on our tasks and create a healthier work–life balance for the future.
